# The neural mechanisms of mindfulness-based pain relief: a functional magnetic resonance imaging-based review and primer

**DOI:** 10.1097/PR9.0000000000000759

**Published:** 2019-08-07

**Authors:** Fadel Zeidan, Jennifer N. Baumgartner, Robert C. Coghill

**Affiliations:** a Department of Anesthesiology, University of California San Diego, San Diego, CA, USA; b Department of Anesthesiology, Cincinnati Children's Hospital Medical Center, Cincinnati, OH, USA

**Keywords:** Mindfulness, Pain, fMRI, Placebo, Meditation

## Abstract

The advent of neuroimaging methodologies, such as functional magnetic resonance imaging (fMRI), has significantly advanced our understanding of the neurophysiological processes supporting a wide spectrum of mind–body approaches to treat pain. A promising self-regulatory practice, mindfulness meditation, reliably alleviates experimentally induced and clinical pain. Yet, the neural mechanisms supporting mindfulness-based pain relief remain poorly characterized. The present review delineates evidence from a spectrum of fMRI studies showing that the neural mechanisms supporting mindfulness-induced pain attenuation differ across varying levels of meditative experience. After brief mindfulness-based mental training (ie, less than 10 hours of practice), mindfulness-based pain relief is associated with higher order (orbitofrontal cortex and rostral anterior cingulate cortex) regulation of low-level nociceptive neural targets (thalamus and primary somatosensory cortex), suggesting an engagement of unique, reappraisal mechanisms. By contrast, mindfulness-based pain relief after extensive training (greater than 1000 hours of practice) is associated with deactivation of prefrontal and greater activation of somatosensory cortical regions, demonstrating an ability to reduce appraisals of arising sensory events. We also describe recent findings showing that higher levels of dispositional mindfulness, in meditation-naïve individuals, are associated with lower pain and greater deactivation of the posterior cingulate cortex, a neural mechanism implicated in self-referential processes. A brief fMRI primer is presented describing appropriate steps and considerations to conduct studies combining mindfulness, pain, and fMRI. We postulate that the identification of the active analgesic neural substrates involved in mindfulness can be used to inform the development and optimization of behavioral therapies to specifically target pain, an important consideration for the ongoing opioid and chronic pain epidemic.

## 1. Introduction

Mindfulness-based meditation practice is associated with promoting a wide-spectrum of health outcomes including anxiety, depression, and pain (see Ref. 22 for thorough review on mindfulness-based clinical interventions). The advent of modern neuroimaging and other objective methodological approaches has substantiated accounts from contemplatives and mindfulness practitioners supporting the purported health promoting benefits of mindfulness-based mental training. We postulate that the scientific field of mindfulness and other mind–body approaches has largely advanced because the health promoting benefits supporting mindfulness are not solely based on subjective reports, but rather, the discovery of the biological substrates supporting such improvements. The present review will delineate a brief, yet comprehensive review of the neural mechanisms supporting pain reductions from the perspective of interindividual variability in dispositional mindfulness, to brief, and long-term mindfulness-based mental training. We will also provide a brief primer to best conduct experiments combining pain-evoking procedures, mindfulness meditation, and functional magnetic resonance imaging (fMRI).

## 2. What is mindfulness?

Mindfulness is defined here as nonreactive awareness of the present moment experience. This construct can be developed and enhanced with mindfulness-based mental training. However, experience with mindfulness meditation is not a prerequisite to being mindful.^24,32^ There are a wide variety of mindfulness-based practices that are subsumed under the general rubric of *“mindfulness meditation.*” Thus, it is critical that the operational specifics supporting mindfulness practices are fully described to promote cross-experimental methodological validity and reliability.^32,72,103^ In the context of the present review, the 2 primary forms of mindfulness practice are characterized as *Shamatha* (focused attention) and *Vipassana* (open monitoring).^72,125^

During focused attention practice, or Shamatha,^125^ the practitioner is taught to sustain attention on an automatic and dynamic stimulus, such as breath sensations, as an analogy for the *qualitative aspects of the present moment.* As attention drifts from the object of focus (eg, breath) to a *distracting* sensory event, the practitioner is taught to acknowledge the event without further reaction, to disengage from the discursive event, and then to return attention to the meditative object (eg, the breath). Shamatha training increases in complexity (eg, mindfulness of breath, emotions, and/or thoughts) as individuals develop expertise at each step. Shamatha practices aim primarily at gaining mental control and stabilization of attention, thus they naturally transition in a relatively undefined manner to open monitoring meditation, or Vipassana practice.

Vipassana meditation has been described as a state of nonappraisal and/or a nonelaborative mental stance.^45,137^ Indeed, there is little to no concrete consensus concerning where Shamatha ends and Vipassana begins. Vipassana practices are associated with an awareness of the awareness (ie, meta-awareness)^37,99^ of arising sensory, affective, or cognitive events. The Vipassana practitioner is said to *experience* each moment without evaluation or elaborative interpretation.

Traditionally, focused attention is taught before, or as a *prerequisite* to, open monitoring practice. The reason for this is likely that a greater capacity for attentional stability and mental control^53,138^ would allow more success in alleviating one's tendency to reflexively engage with, and appraise, ongoing experience. As the practitioner becomes more adept in meditation, he/she will be able to use a unique cognitive approach where consciously available sensory events can be simply “let go,” presumably leading to significant reductions in affective or cognitive appraisals/reactions to the event. Said another way, the practitioner “watches” experience as it unfolds over time without cognitive elaboration or emotional reactivity. As skills in mindfulness meditation continue to develop, the practitioner brings mindfulness more effortlessly into meditation and other aspects of everyday life.^59^ It is postulated that the state transitions to a trait as a function of practice frequency and neural plasticity. However, the cultivation of mindfulness does not exclusively require training in mindfulness meditation. Some individuals are simply more “mindful” than others, as evinced by higher scores on standardized measures of dispositional/trait mindfulness without any formal training in meditation.^24,52,128,142^

## 3. Why mindfulness for pain?

Given that pain is instantiated by a highly distributed network of brain mechanisms,^21^ treatments that work in a highly focal fashion on one neurotransmitter system or on one brain region or pathway are likely to be of limited efficacy. Treatments that can target multiple nodes within this distributed network are crucially needed. Mindfulness may have that ability because it uniquely enhances cognitive control,^138^ emotion regulation,^73^ acceptance (ie, nonreactivity),^68,92,116^ and improves mood.^17,36^ There is also a great deal of insight from Buddhist contemplatives in the way that mindfulness-based mental training impacts the subjective experience of pain. The *Sallatha Sutta*, a Theravadin Buddhist scripture from the Pali Canon (translated as *The Arrow or The Dart*), explicitly states that there is a key difference in how mindfulness trained and “untrained” individuals experience pain.^3^ The Sutta analogizes the reaction to pain to being stung by an arrow and, in the case of untrained individuals, being struck immediately after by a second arrow. The 2 arrows are described as representing physical and mental pain, respectively. The first arrow could refer to the noxious sensation one initially feels, while the second dart could be described as the worry, distress, and pain-evoked suffering that follows. The claim of Buddhist contemplatives is that mindfully trained individuals, because they do not cling to sensory pleasure, also do not engage cognitive and affective appraisals of pain.

## 4. Does mindfulness meditation relieve chronic pain?

Chronic pain affects more than 100 million Americans, 1.5 billion people worldwide, and costs the United States an estimated $635 billion per year in medical expenses and lost work productivity.^33^ Despite treatment advances, the pervasiveness and burden of chronic pain has dramatically increased Medicare expenditures for steroid injections (↑629%) and opioid treatments (↑423%).^31^ The widespread use of opioids to alleviate chronic pain has led to the so-called “opioid epidemic”^84^ with an exponential rise in opioid misuse and addiction.^50,98^ These staggering statistics signify the importance of developing fast-acting nonpharmacologic approaches to treat acute exacerbations of chronic pain. To this extent, the need for effective nonpharmacological treatment options has spurred much interest in mind–body approaches toward mitigating chronic pain. Mindfulness meditation-based interventions improve chronic pain symptomology across a wide range of pain conditions, including fibromyalgia,^29,48^ headache disorders,^130^ chronic pelvic pain,^38^ irritable bowel syndrome,^41,44^ and chronic lower back pain^19,56,58,82,83^—the most prevalent and financially burdensome chronic pain disorder. Research suggests that mindfulness meditation improves chronic pain symptomatology through unique mechanisms, such as disengagement from pain-related threat,^119^ extinction of fear conditioning, acceptance-based coping strategies,^81^ and strengthening one's ability to self-regulate affective appraisals of nociceptive input.^42^

Much of the work on the efficacy of mindfulness-based interventions for the treatment of chronic pain has focused on outcomes associated with mindfulness-based stress reduction (MBSR) and its varieties.^56,57^ Mindfulness-based stress reduction is a structuralized, group-based psychosocial intervention consisting of mindfulness meditation, yoga, and cognitive-behavioral therapy with the goal of integrating mindfulness into everyday life. In their seminal work, Kabat-Zinn et al.^56^ showed that a 10-week MBSR program reduced pain reports, pain-related behaviors and improved general vitality in chronic pain patients, effects that were maintained at 15-month follow-up.^58^ However, this initial work lacked a control group, and thus, while promising, subsequent research was warranted. A more recent study showed that MBSR produced greater reductions in pain catastrophizing in chronic low-back pain patients compared with cognitive-behavioral therapy (CBT) and standard of care.^118^ It is interesting that MBSR outperformed CBT given the explicit focus of CBT on controlling maladaptive thoughts and behaviors, suggesting that mindfulness training may uniquely buffer against future-oriented, catastrophic thinking. In fact, growing research suggests that the nonreactivity component of mindfulness is negatively related to pain catastrophizing.^30^ However, the long-term benefits of MBSR on pain catastrophizing are questionable because group differences were no longer significant at follow-up,^118^ implying that training should to be continued to facilitate lasting changes in the propensity to catastrophize. By contrast, in a seminal study, Cherkin et al.^19^ found that both MBSR and CBT significantly improved ratings of functional limitations, pain-related distress, and pain intensity in chronic lower back pain patients when compared with standard of care. In the same study, findings persisted for MBSR and CBT at 8-, 26-, and 52-week follow-up, demonstrating the durability of mindfulness on certain pain-related outcomes over time. Taken together, this growing body of work demonstrates that a few weeks of mindfulness training improves pain and health outcomes in people suffering from chronic pain, and with some evidence of persistent benefit. Yet, the precise neural mechanisms supporting pain relief by mindfulness meditation remains relatively uncharacterized.

## 5. Multiple neural mechanisms supporting mindfulness-based pain reductions

Electrophysiological (EEG) and brain imaging methods have provided an important avenue for elucidating the neural mechanisms supporting the modulation of pain by mindfulness meditation. Converging lines of evidence demonstrate that mindfulness-based analgesia engages distinct neural processes that vary across training level. Here, we will delineate the neural mechanisms supporting mindfulness-based pain modulation across no to brief to long-term training levels. We will focus primarily on studies using fMRI.

### 5.1. Brain mechanisms supporting the relationship between higher trait mindfulness and lower pain sensitivity

**Figure 1.**
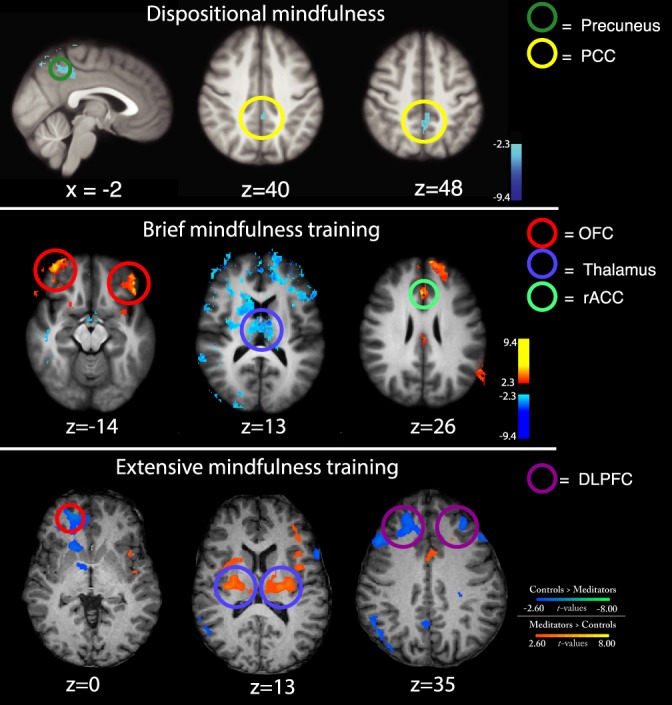
First row (dispositional mindfulness): Greater deactivation of the posterior cingulate cortex (PCC)/precuneus was associated with higher trait mindfulness (Freiburg Mindfulness Inventory) and lower pain during noxious heat stimulation.^142^ Second row (brief mindfulness training): Higher mindfulness meditation-induced activation of the bilateral orbitofrontal cortex (OFC), rostral anterior cingulate cortex (rACC), and greater thalamic deactivation was associated with greater pain relief during noxious heat.^141^ Third row (extensive mindfulness training): When compared with age-matched controls, adept Zen meditators exhibited significant decoupling in low-level pain-related brain activation (thalamus) and brain regions that process appraisals and affect (medial OFC [mOFC] and dorsolateral PFC [DLPFC]) during noxious heat stimulation.^45^

Individuals that have never practiced mindfulness meditation may still be highly mindful as a function of genetics, environment, psychosocial, and cultural factors. *Trait or dispositional mindfulness* is the innate propensity to be aware of the present moment in a nonreactive manner.^27^ Trait mindfulness can also be developed and increased through mindfulness-based mental training. Individuals who are low on the trait mindfulness spectrum exhibit higher pain in a number of clinical pain populations.^81,90,100^ Two recent studies combined psychophysics, noxious heat stimulation, and distinct functional neuroimaging techniques to identify the neural mechanisms supporting the postulated relationship between trait mindfulness and pain sensitivity^142^ and pain threshold.^52^ Zeidan et al.^142^ used perfusion-based arterial spin labeling (ASL) fMRI to determine the significant relationship between higher dispositional mindfulness (as measured by the Freiburg Mindfulness Inventory) and lower pain ratings in response to noxious heat (49°C).^142^ As hypothesized, higher trait mindfulness was associated with greater deactivation of the posterior, midline nodes of the so-called default mode network. The default mode network is defined by oscillating activity within a group of distinct brain regions (medial prefrontal cortex, posterior cingulate cortex [PCC]/precuneus, inferior, and lateral temporal cortices) and is associated with facilitating self-referential processes.^28,76,91,107^ Specifically, greater deactivation of the dorsal PCC and precuneus was associated with higher trait mindfulness, and greater activation of the dorsal PCC during noxious heat was associated with higher pain reports during noxious heat (Fig. 1).^142^ In a similar study employing functional connectivity analyses with blood oxygen level-dependent (BOLD) signaling fMRI, Harrison et al.^52^ found that greater trait mindfulness (measured by the Five Facet Mindfulness Questionnaire) was associated with higher pain threshold values. Higher trait mindfulness was associated with weaker connectivity between the central nodes of the default mode network (ie, medial prefrontal cortex, PCC/precuneus) and stronger connectivity between the precuneus and somatosensory cortices. These findings are remarkably consistent with the principles of mindfulness supporting the disposition to attend to immediate sensory aspects of experience and the ability to disengage from immediate appraisals and corresponding ruminations. Taken together, these 2 studies provide unique mechanistic insight that trait mindfulness is associated with lower pain and lower PCC/precuneus processing, suggesting that trait mindfulness is associated with a lower propensity to ruminate on^65^ and personalize^4^ the appraisal of arising noxious sensory events.^142^ Furthermore, individuals who exhibit higher trait mindfulness may have a greater ability to decouple noxious sensory discriminations from affective appraisals.^142^ This is important because even brief bouts of mindfulness training (less than a week) can significantly increase dispositional mindfulness on average by 13%.^135,138,139,141^ These findings could assist us to better develop and tailor adjunctive pain therapies to specifically target reductions in PCC/precuneus and increase trait mindfulness activity to treat pain.

### 5.2. Neural and physiological processes supporting mindfulness-based pain relief after brief mental training

Longitudinal and carefully controlled studies examining the effects of *brief* mental training regimens can provide insight into measuring explicit changes in behavioral and physiological pain responses. To this extent, it is now well established that mindfulness meditation significantly reduces pain after brief mental training (4 days > 4 weeks).^62,134–136,141^ In one of the first preliminary studies to examine the effects of mindfulness meditation on pain, Kingston et al.^62^ found that 6 hours of mindfulness-based mental training was associated with higher pain tolerance during the cold pressor test, when compared with an active, visual imagery-based (“imagine walking through a garden”) control condition. The authors reported no significant between-group differences in mindfulness (as measured by the Kentucky Inventory of Mindfulness Skills), blood pressure, or mood.^62^

Others have postulated that mindfulness-induced pain relief engages mechanisms supporting divided attention, relaxation, and/or positive mood.^15,55^ To better address this issue, Zeidan et al.,^138^ across 3 separate experiments, dissected the pain relieving effects of mindfulness meditation regimen (three 20-minute Shamatha + Vipassana training sessions) from a robust, math distraction and relaxation condition during noxious electrical stimulation. Mindfulness meditation during noxious electrical stimulation significantly reduced pain responses above and beyond analgesia observed during math distraction and relaxation. Surprisingly, mindfulness meditation training significantly increased pain threshold when compared with before the intervention and the control group.^138^ These findings provided credence that mindfulness-based pain relief may engage unique physiological mechanisms from other cognitive techniques to reduce pain.

In their follow-up study, Zeidan et al.^141^ examined the behavioral and neural mechanisms supporting state mindfulness-induced pain relief before and after a brief, mindfulness training regimen (four 20-minute sessions) during noxious heat (49°C) in healthy participants. In the pre- and post-fMRI sessions, participants were instructed to “begin meditating and to focus on the changing sensations of the breath” during noxious heat- and perfusion-based fMRI (ASL). Focusing on the breath *before* the intervention reduced pain unpleasantness ratings but not pain intensity ratings when compared with rest. After training, mindful attention to the breath significantly reduced pain intensity (↓40%) and pain unpleasantness (↓57%) ratings when compared with rest. Mindfulness meditation produced widespread activation of the (1) ACC extending from the dACC to the perigenual ACC (pgACC), (2) bilateral orbitofrontal cortex (OFC), (3) the primary somatosensory cortices (SI) corresponding to the face region, (4) bilateral ventral striatum, and (5) deactivation of the default mode network.^141^ In the presence of noxious heat, mindfulness significantly reduced SI activation corresponding to the stimulation site (right leg), suggesting that mindfulness reduces ascending nociceptive inputs to somatosensory cortical regions. Regression analyses revealed that greater pain relief was directly associated with greater activation of the pgACC, right anterior insula, and bilateral OFC. Greater mindfulness-based pain relief was also associated with significant extensive, bilateral deactivation of the thalamus, a critical node of ascending nociceptive information from the spinal cord (Fig. 1).^49^ These findings are particularly consistent with the principles of mindfulness. That is, the pgACC facilitates top-down regulation of negative effect, affective modulation of pain, and autonomic regulation.^11,16,101,120,121^ The right anterior insula has been repeatedly implicated in processing interoceptive awareness and regulation of nociception.^20,21,26,64,86,109,111^ The OFC, a highly evolved brain region, is associated with increasing positive mood and altering the contextualization of arising sensory events.^35,85,88,94^ We propose a working theoretical framework that connects mindfulness-based pain relief with multiple, neural mechanisms that support both cortico-cortical and cortico-thalamo-cortical interactions.

Randomized, placebo-controlled studies are characterized as the “gold standard” to defining the effectiveness and active novel pain relieving treatments. Yet, placebo-controlled meditation studies have been very limited. This is problematic when considering that meditation is very susceptible to placebo responses and nonspecific effects such as slow breathing, demand characteristics, beliefs related to practicing meditation, conditioning, posture, among others. Thus, in the follow-up experiment, Zeidan et al.^135^ examined if the physiological and neural (ASL fMRI) mechanisms supporting mindfulness-based pain relief are distinct from those engaged by different placebo conditions. Healthy, pain-free participants were randomly assigned to 1 of 4 (four 20-minute/session) training regimens: (1) a mindfulness meditation akin to one previously described, (2) a placebo cream conditioning, (3) a sham mindfulness meditation, and 4) a book-listening control. Participants in the placebo-conditioning group were led to believe that the effects of an experimental form of Lidocaine was being evaluated, in which the pain-relieving effects of the cream (petroleum jelly) progressively increased with repeated application. Participants were led to believe that the cream was analgesic by covertly reducing stimulus temperature after application of the cream in each conditioning session. The sham mindfulness regimen mirrored mindfulness meditation on all nonspecific and psychosocial factors (posture, breathing, and eyes closed), but without the explicit training on focusing on the changing sensations of the breath in a nonreactive fashion. Thus, this group (theoretically) allowed us to disentangle the active mechanisms that were specific to *mindfulness.* All conditions significantly reduced pain intensity and pain unpleasantness ratings compared to the book-listening controls. Mindfulness meditation was more effective than placebo cream, placebo (sham) mindfulness, and control conditions at reducing pain ratings. The neural mechanisms supporting mindfulness-based pain relief were, again, found to be associated with greater activation in the OFC, pgACC, and right anterior insula.^135^ There was also significant deactivation of the PAG and thalamus, replicating and extending on the findings described in the previous ASL study.^141^ Placebo cream-induced pain relief was associated with significant reductions of contralateral, nociceptive somatosensory areas (ie, parietal operculum and secondary somatosensory cortices [SII]) and greater activation of the dorsolateral prefrontal cortex [DLPFC]), likely reflective of perceive controllability of pain^5,97,102^ and lower expectations for pain.^6,64^ This was the first ASL fMRI placebo experiment and largely replicated the findings supporting other pain-placebo–focused brain imaging studies.^11,14,63,64,70,122,123^ Regression analyses revealed that sham mindfulness induced pain relief was associated with lower respiration rate. These findings provide clear mechanistic distinctions between mindfulness and placebo. As described earlier, mindfulness engages multiple mechanisms supporting top-down regulation of pain. By contrast, sham mindfulness meditation likely employs bottom-up processes. Taken together, these findings provide robust evidence that the state effect of mindfulness meditation, after brief mental training, significantly reduces pain and recruits multiple, distinct neural processes from robust placebo conditions.

## 6. Neural mechanisms supporting pain relief after extensive mindfulness meditation experience

Research employing individuals with extensive mindfulness meditation experience (>1000 hours of practice) has provided insight into the ability of adept practitioners to evoke more stabilized, state- and trait-level changes in nociceptive processing, and corresponding pain responses. There is reliable and growing evidence demonstrating that long-term mindfulness practice can lead to significant increases in pain threshold values and lower pain sensitivity even when participants are not explicitly practicing mindfulness.^13,19,43,46,82,83^ Grant and Rainville^46^ were the first to demonstrate that adept Zen practitioners (a mindfulness practice similar to Vipassana) required significantly higher noxious heat stimulation to report the same level of pain as age-matched controls. In a follow-up fMRI (BOLD) study using a functional connectivity analyses during noxious heat stimulation and overlapping participant sample, Zen practitioners exhibited a significant *decoupling* between brain regions supporting nociceptive processing (thalamus; insula; SII) and sensory/affective appraisal (dlPFC; dACC) when compared with age-matched controls (Fig. 1).^45^ Greater decoupling between these brain regions was associated with higher pain tolerance in the Zen group. The authors postulated that the Zen practitioners preserved the capacity to attend to sensations but reduced affective appraisals of said sensory events. Importantly, practitioners were not meditating in this study, potentially providing credence of longstanding Buddhist accounts implicating extensive mindful practice with pronounced, trait-like changes in subjective experience and perception. Another study examined the state effects of mindfulness on pain responses and corresponding brain mechanisms (BOLD fMRI) in long-term Vipassana practitioners and demographically matched meditation naïve controls.^40^ Practicing mindfulness in response to 1-second noxious electric stimuli reduced pain unpleasantness (↓22%) and anticipatory anxiety (↓29%) ratings when compared with rest and control conditions. Surprisingly, mindfulness meditation did not significantly reduce pain intensity ratings when compared with controls. Mindfulness-based pain relief was associated with decreased activation in the lateral PFC and increased activation in the right posterior insula/SII.^40^ The authors postulated that this reflects “decreased cognitive control.” One could also take the perspective^132,137,143^ that mindfulness was associated with enhanced cognitive flexibility, potentially reflected by observed increases in rACC during meditation. Other groups^45,114^ have also postulated that it requires a significant degree of cognitive-affective control to reduce appraisals (↓PFC) of ascending noxious sensory events.

To this extent, one of the defining features of mindfulness meditation is the emphasis on nonevaluative awareness of present moment experience. It can reasonably be construed, then, that this experiential acceptance should reduce expectations of impending noxious stimuli that could exacerbate the pain experience.^64,140^ Lutz et al.^71^ tested this postulation by examining the influence of long-term mindfulness meditation (>10,000 hours of practice in the Nyingma and Kagyu traditions of Tibetan Buddhism) on psychophysical and neural responses to noxious heat stimulation. Control participants were nonmeditators that were instructed to practice mindfulness at home for 1 week. Surprisingly, both adept and novice meditators reduced pain intensity ratings during Shamatha practice and noxious heat, and there were no significant group differences. Vipassana-based meditation produced significant pain unpleasantness reductions in the adept meditators when compared with novices. The adept meditators exhibited less activation in the contralateral anterior insula and the rostral aspect of the midcingulate cortex during the prestimulus period when compared with controls. Furthermore, the greater the meditative experience, the greater the deactivation of said regions. Brown and Jones^13^ used electroencephalography (EEG) and pain-evoking laser stimulation to examine behavioral and EEG pain responses in adept meditators. Similar to Lutz et al., they found that lower pain responses were associated with significantly lower anticipatory event-related potentials in the inferior parietal and midcingulate cortex in the meditation but not control group.^13^ Greater meditative experience also predicted lower anticipatory EEG responses and greater pain relief. Remarkably, these effects were detected during a nonmeditative state and provided a clear scope of the trait-like pain modulatory changes that can occur with extensive mindfulness training.^13^ Finally, in a recent preliminary study, Taylor et al.^115^ recruited 11 adept meditators (>1000 hours of training in the Zen, Bodhicitta, and Kadampa tradition) and 51 control subjects to examine whether mindfulness reduces pain and anticipatory skin conductance reactions, specifically the nociceptive flexion reflex, in response to a fear condition task and noxious electrical stimulation. They found that the meditation group exhibited less pain sensitivity and reduced fear-induced pain facilitation than controls. Meditators and nonmeditators displayed comparable nociceptive flexion reflexes, suggesting that mindfulness does not (1) modulate nociceptive processes at the level of the spinal cord, (2) engage *classical* descending inhibition of pain mechanisms, and (3) reduce anticipatory responses corresponding to conditioned pain-focused fear response, but rather direct expectations of impending stimuli, a finding consistent with previous work.^13,71^ The authors further speculated that reductions in fear-conditioned pain learning might be a mechanism by which mindfulness reduces the progression of acute to chronic pain. Taken together, these mechanistic findings^13,71,115^ show that attenuating the expectations of impending sensory events by sustaining attention in the present moment are directly associated with facilitating mindfulness-based pain relief.

## 7. The role of endogenous opioidergic systems (or lack thereof) in mindfulness-induced pain relief

A plethora of endogenous neurotransmitters, including the cannabinoid, serotonergic, dopaminergic, and opioidergic systems, at least, are partially engaged in the cognitive modulation of pain.^7,127^ To date, the endogenous opioidergic system is characterized as the primary endogenous pain modulatory system.^127^ Higher order brain regions (PFC; rACC) project to the PAG, the rostral ventral medulla, and the dorsal horn of the spinal cord to reduce the elaboration of spinal and supraspinal nociceptive processing through the release of endogenous opioids^9,77,78^ that largely bind with mu, delta, and kappa receptors.^54,69^ Pain relief produced by placebo,^2,34,47,67,147^ acupuncture,^51^ conditioned pain modulation,^61^ distraction,^108^ and hypnosis^39,110^ is meditated, in part, by endogenous opioidergic systems.

Mindfulness-based analgesia, after brief mental training, is associated with greater activation of the rACC, OFC, and insular cortices, brain regions that contain high concentrations of opioid receptors.^18,117,124^ However, mindfulness meditation also deactivates the PAG, a central node in opioidergically mediated descending inhibition of pain.^7^ To bridge this exploratory gap, Zeidan et al.^134^ conducted a double-blinded, randomized study of healthy, meditation-naïve participants to determine whether mindfulness pain relief engages endogenous opioids in response to intravenous administration (IV) of placebo saline and high-dose naloxone, the opioid antagonist, when compared with a book-listening control. In brief, mindfulness, after brief mental training, effectively reduced behavioral responses in the presence of opioid blockade and saline, demonstrating that mindfulness-based analgesia is not mediated by endogenous opioids. As a supplementary query, one could postulate that mindfulness-based pain relief, after extensive mindfulness training, could engage endogenous opioidergic systems due to progressive development of opioidergically driven processes such as conditioning, expectations, and other placebo-type mechanisms. Using a double-blinded, cross-sectional design, May et al. (2018) assessed the role of endogenous opioids in mindfulness-based pain relief in adept mindfulness practitioners with extensive training (>4,000 hours of practice). Surprisingly, when compared with saline and the control group, high-dose IV naloxone infusion produced *greater* pain relief in the meditation group, providing further evidence that mindfulness across training levels produces analgesia independent of endogenous opioids.^79^ We postulated that expert meditators may have exerted more effort in the presence of naloxone to counteract the effect of naloxone, and that other nonopioidergic systems^10^ may have been engaged to compensate for opioid blockade.^89^ By contrast, another group used a randomized, crossover design and reported that mindfulness-based pain relief was reversed by naloxone^105^ in response to a brief (10 seconds) noxious cold stimulus. In our rebutting commentary of this study, we postulated that the authors' interpretations were not justified because of the significant decrease in pain (ie, ↓19%) during mindfulness and naloxone infusion and the comparable reductions during meditation and saline infusion (ie, ↓25%).^133^ Importantly, the authors did not directly statistically compare the differences in pain between the saline and naloxone conditions, which begs the question on why they used the elegant crossover-controlled design. The authors go on to say in their rebuttal to our commentary^104^ that “experience seems to be a crucial factor in opioid involvement in mindfulness meditation.” Our recent study demonstrating that mindfulness-based analgesia is more pronounced during naloxone in long-term meditators (mean experience >4000 hours of meditation practice) potentially invalidates this interpretation.^79^ Taken together, these findings are important because they provide supplementary evidence that mindfulness engages unique mechanisms to reduce pain that are distinct from those mediated by placebo analgesia (ie, endogenous opioids; ↓ PAG). Because opioid and nonopioid processes synergistically interact with reduce pain, these findings suggest that mindfulness could be used in conjunction with traditional pain therapies to optimize analgesia, an important finding for the millions of chronic pain patients seeking effective, self-regulatory pain therapy.

## 8. A brief primer: functional neuroimaging of mindfulness and pain

The widespread employment of functional neuroimaging methodologies and identification of the health promoting mechanisms supporting meditation is credited with the explosive proliferation of mindfulness-based research. Functional magnetic resonance imaging, positron emission tomography (PET), and ASL fMRI have provided means to collect direct (perfusion-based fMRI; PET) and indirect (BOLD; fMRI) measures of cerebral blood flow (CBF), which provide higher spatial resolution (ie, indicating where neural activity may be occurring), although this comes at a tradeoff, with poorer temporal resolution (2–5 seconds) than EEG/event-related potential. Here, we will provide very brief suggestions on how to best conduct mindfulness-pain research in combination with fMRI.

### 8.1. Arterial spin labeling or blood oxygen level–dependent functional magnetic resonance imaging to study state mindfulness and pain?

Both ASL and BOLD fMRI examine CBF to make inferences about neural activity. There are a number of reasons that we propose that perfusion-based, ASL fMRI method is more appropriate for examining the effects of *state* mindfulness meditation when compared with BOLD fMRI. Arterial spin labeling fMRI provides a fully quantifiable and direct measurement of CBF (mL/100 g of tissue/min). By contrast, BOLD is an indirect surrogate of CBF that assesses blood oxygen in arbitrary units. When compared with BOLD fMRI, ASL is less sensitive to low-frequency drifts in signal intensity that can occur as an individual's body *relaxes* as a function of time or as the scanner heats up over the course of an imaging series.^126^ Accordingly, BOLD fMRI is not well suited to appropriately image task-related brain activation longer than 30 seconds.^1^ Furthermore, when compared with BOLD, ASL fMRI can reliably assess rostral neural regions, such as the OFC,^126^ a brain area repeatedly implicated in mindfulness meditation,^135,141^ but suffering from substantial susceptibility artifacts. Nevertheless, BOLD fMRI does enjoy higher signal-to-noise ratio when compared with ASL.^87^ Thus, the utility of ASL vs BOLD comes down to the primary aims of the study.

### 8.2. State mindfulness produces respiration-induced functional magnetic resonance imaging artifacts

As fMRI scanners increase their respective field strengths, physiological noise has become a significant confound reducing the applicability and generalization of functional neuroimaging findings.^93^ This is an important caveat because state mindfulness meditation reliably (1) reduces respiration rate and, in parallel, decreases CBF due to lower arterial levels of carbon dioxide^60,131^ and (2) alters chest movements that cause magnetic field changes, rendering a physiological artifact.^112^ This is problematic because meditation-induced changes in breathing are directly task-specific and can dramatically misconstrue the interpretation of findings. The changes in breathing are meditation-specific, and the brain (de) activations could be interpreted as meditation-related mechanisms, when they are potentially due to physiological noise.^12,96^ In our laboratory, we have seen (*unpublished findings*) significantly greater white-matter activation when compared with gray matter during meditation practice. One can make the mistake of regressing out each individual's respiration rate from his or her respective fMRI analysis. However, that would likely remove neuronal activation that directly corresponds to mindfulness-based breath focus, as opposed to controlling for physiological noise. Furthermore, the employment of perfusion fMRI may not directly correct for physiological artifacts, although it is better suited to identify if CBF deviations occurred (ie, ASL produces fully quantifiable CBF measurements). Thus, it is critical that researchers evaluate and correct for this issue by (1) collecting respiration data, (2) visually inspecting data for significantly greater white-matter activation when compared with gray matter, (3) segmenting and comparing white-matter and gray-matter brain, and (4) inputting segmented white values as covariates of no interest in the statistical analyses using a component-based noise correction method (CompCor; temporal or anatomical variants).^8^

### 8.3. ON/OFF block functional magnetic resonance imaging designs are not appropriate to examine state mindfulness

Functional neuroimaging experiments use “ON/OFF” block designs to identify brain activation corresponding to any specific event (eg, noxious stimulation; cues; appraisals corresponding to a stimulus, etc.). For example, a traditional BOLD-based fMRI experimental paradigm may be designed to contrast brain activation in response to 10-second plateaus of noxious, 49°C and innocuous 35°C heat, respectively. The 49° stimulus is generally characterized as the “on” and 35°C as the “off” condition, respectively. Because this hypothetical experimental paradigm controls for contact of the thermal probe and change in stimulus temperature, significant brain activation could be interpreted as brain activity corresponding to 49°C when compared with 35°C, and vice versa. However, this on–off paradigm cannot be used to measure state mindfulness. That is, directing subjects to meditate for 10 seconds (ON) and rest (OFF) for 10 seconds will lead to contamination of the “OFF” period because the cognitive state of mindfulness meditation cannot be “turned off and on” like a thermal stimulus. There are carryover effects from mindfulness meditation that can last minutes to days. Thus, we propose starting the experiment with rest (ie, nonmeditation) for the first half of the experiment and instructing subjects to meditate in the second half of the study. This paradigm is weakened by obvious order effects, but using a paralleling nonmanipulation group/condition can easily control this for experimental confound. It is important to note that these recommendations are explicit to state mindfulness meditation activation studies. It is perfectly acceptable to use BOLD fMRI-based resting state functional *connectivity* analyses to measure stabilized changes in neural functioning because ASL fMRI is not yet optimized for connectivity studies.

### 8.4. Strong control conditions are critical to disentangle mechanisms supporting mindfulness-based analgesia

The neural mechanisms supporting mindfulness-based practices still remain poorly characterized.^113^ Mindfulness-based pain relief is believed to be associated with a spectrum of nonspecific factors such as distraction, slow breathing, placebo, conditioning, facilitator attention, social support, body posture, relaxation, and demand characteristics. This is a limitation in fostering the validity of this practice to relieve pain. We suggest that mindfulness-pain neuroimaging studies should use comparison conditions that most closely resemble mindfulness practices and suited to disentangle the mechanisms that are postulated to be engaged by mindfulness. To better address this issue, we recently developed and validated a sham-mindfulness meditation comparison condition.^135,139^ Sham-mindfulness meditation (ie, placebo mindfulness) was designed, so that the primary difference between the mindfulness and sham-mindfulness practice was the explicit instructions corresponding to the nonreactive attention to the breath sensations used in the mindfulness meditation. Participants were first instructed that they were randomly assigned to the mindfulness meditation group, and that mindfulness training was secular. In each of the training sessions, subjects were instructed to close their eyes, and to take a deep breath “as we sit here in meditation” every 2 to 3 minutes.^135,139^ All other aspects of the sham-mindfulness meditation intervention (ie, posture, training room, facilitator; time spent providing instructions; and eyes closed) matched the mindfulness meditation-training regimen. We also paralleled the fMRI-session's experimental directives (“begin practicing mindfulness meditation until the end of the experiment”) across the mindfulness and sham-mindfulness groups.

Other appropriate fMRI controls for mindfulness include divided attention tasks,^55,106,136^ relaxation conditions,^136^ cognitive-behavioral therapies, and techniques that may share mechanisms that may be engaged by mindfulness (ie, slow breathing manipulations). Some recent studies have successfully disentangled the active mechanisms supporting mindfulness-based health promotion. Creswell et al.^23^ compared behavioral, inflammatory stress [ie, interleukin-6 (IL-6)] and neural mechanisms supporting an intensive 3-day mindfulness meditation and 3-day health enhancement relaxation program in unemployed and clinically stressed adults. All aspects of the relaxation program were matched to the mindfulness regimen intervention (including time of the interventions held on the same weekends and even the location of the intervention). Mindfulness-induced stress reductions were associated with stronger functional connectivity between the PCC and dlPFC, mechanisms supporting executive-level control of emotions. By contrast, the relaxation group exhibited higher IL-6 responses after the intervention. Another group used a health enhancement program (HEP) to disentangle the mechanisms supporting mindfulness.^74,75,95^ The HEP controlled for facilitator allegiance, social support, and other components related to participating in a mindfulness program. Although the HEP and mindfulness conditions were effective at improving mood, stress, and cognition, they did not significantly differ. However, the mindfulness program was more effective at reducing inflammatory markers in response to a social stress test^95^ and pain.^74^ Taken together, these findings demonstrate that some mindfulness techniques are not more beneficial than active controls on some health outcomes (stress and cognition) but superior on other outcomes (pain). Nevertheless, they demonstrate that mindfulness engages unique health-promoting mechanisms that are important to better optimize the development and tailoring of said interventions for different patient populations.

### 8.5. Mindfulness training for the scanner

In light of the ongoing opioid epidemic, nonpharmacological pain therapies such as mindfulness-based approaches will continue to be examined by combining subjective reports and functional neuroimaging methodologies. We have provided a number of supplementary suggestions that may aid in conducting mindfulness-pain fMRI studies. Practicing mindfulness meditation is quite difficult to perform in a quiet room. Mindfulness practice difficulty is exacerbated in the fMRI environment. The scanner (1) is very loud, (2) confines the individual, and (3) renders the participant unable to practice mindfulness in an upright, seated position. To address this, we and others^66^ have conducted mindfulness training while subjects are lying down and during an audio recording of the scanner sounds. We initially begin our mindfulness training regimens while subjects are in a more traditional pose (ie, sitting *up* in a chair) and then instruct individuals to lie in the supine to practice with the sounds of the scanner (after the first, 3 training sessions). We also recommend conducting mindfulness training in a “mock scanner” or a similar environment to better control for claustrophobic individuals and to better prepare subjects to meditate in the scanner.

## 9. Considerations and future steps for mindfulness-based pain research

Here, we show that mindfulness meditation-based pain relief does not engage one particular brain mechanism to reduce pain, but rather a combination of processes that are reflective of effortful, reappraisal processes. In light of Francis Crick's “speculative…searchlight hypothesis,”^25,80^ we propose that the observed relationship between mindfulness-based pain relief and thalamic deactivation reflects a remote tuning mechanism that is facilitated by feedback connections between excitatory, glutamatergic OFC innervations on the thalamus and subsequently the inhibitory, GABAergic thalamic reticular nuclei (TRN).^144–146^ Coincidently, these neuroanatomical projections (OFC > TRN) reflect a gating mechanism of ascending sensory information that selectively modulates, through higher order cognitive control processes (ie, focusing on breath sensations), sensory and emotional salience of said sensory events to significantly reduce the thalamic transmission to early somatosensory cortices (ie, SI). This mechanism is critically involved in integrating real-time sensory and affective information,^129^ assessing value,^49^ and regulating “passage to the cortex”.^146^ Unfortunately, ASL fMRI does not provide optimized means to directly test this hypothesis using functional connectivity analyses, due to low signal-to-noise resolution. Nevertheless, these findings demonstrate that mindfulness-based pain relief engages multiple brain mechanisms that are relatively distinct from other cognitive manipulations. Importantly, the Zeidan et al. (2011) study lacked a matched and active control group, and thus, it was difficult to explicitly ascertain whether mindfulness reduces pain through brain mechanisms that are reflective of placebo analgesia.

The postulated mechanisms supporting mindfulness-based pain relief may not explicitly relate to eradicating the intensity of pain but suggest that mindfulness alters the contextual evaluation of innocuous *and* noxious sensory events. Mindfulness meditation, across all reported pain focused studies, impacted the affective dimension of pain more so than sensory pain. However, more brain imaging research is needed to clearly extrapolate the mechanisms that mindfulness impacts chronic pain, as the large proportion of said fMRI-mindfulness studies were conducted in healthy individuals. To this extent, it will no doubt be fruitful to incorporate PET studies to better identify neurochemical-based interactions supporting mindfulness-based analgesia. This line of research would, if properly designed, provide a clear understanding of the different classes of neurotransmitters involved in mindfulness and provide another means of testing our working hypothesis on the role of the TRN in modulating pain. Mindfulness is susceptible to nonspecific effects, thus we highly recommend appropriate placebo-based comparisons to better isolate genuine mindfulness mechanisms. We also need to better characterize the neural mechanisms supporting mindfulness-based pain relief in specific chronic pain populations. For instance, the neural processes supporting pain relief in chronic low-back pain may differ from those found to be effective at treating migraine pain. We also need to appreciate how long the effects of meditation last and how much training is required. Nevertheless, it is clear that mindfulness effectively reduces pain and does so by engaging processes that reflect changes in one's *relationship* with respective pain. This is important for those seeking ways to reduce the impact of developing chronic pain and the common comorbidities (anxiety and depression) that exacerbate the pain experience. We predict that the mechanisms supporting mindfulness-based pain relief become more robust with more practice, a distinction from traditional pain therapies that exhibit increasing tolerance and plateaus in efficacy. When considering the ongoing chronic pain and opioid epidemic, the utility of mindfulness meditation may prove to be an important tool to teach individuals to self-regulate pain directly with a present-centered and acceptance-based strategy.

## Disclosures

The authors have no conflict of interest to declare.
